# Supplementary material for: The interactome of intact mitochondria by cross-linking mass spectrometry provides evidence for coexisting respiratory supercomplexes
Source: Mol Cell Proteomics. 2017 Dec 8;17(2):216–32. doi: 10.1074/mcp.RA117.000470 (PMC5795388; doi:10.1074/mcp.RA117.000470)
Supplement: Supplemental Data [file supp_RA117.000470_133922_0_supp_23978_fzffwf.zip › spectra_annotation/mito_spectra_annotation/451-1-8-1-33-1.pdf]

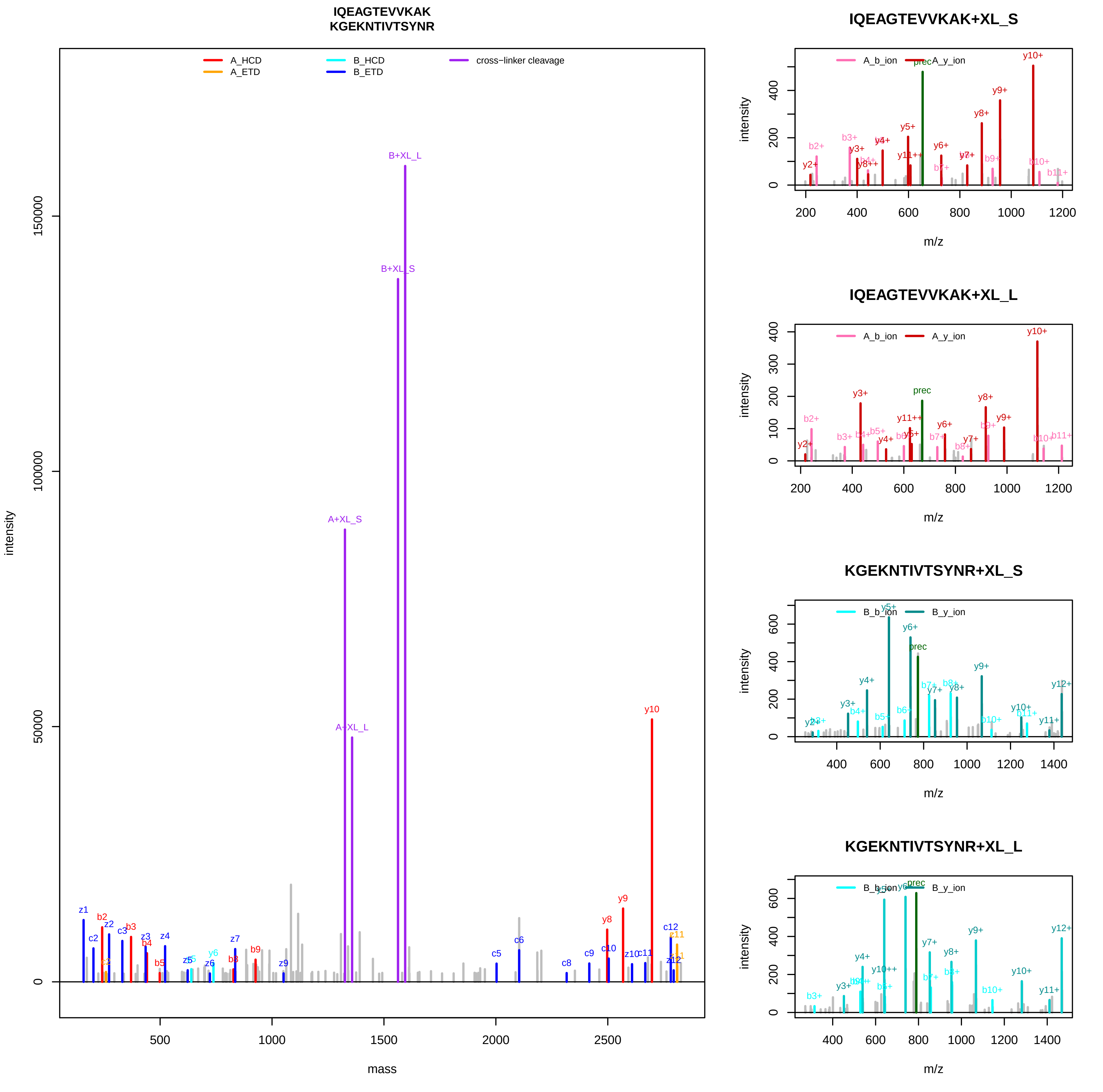
IQEAGTEVVKAK
KGEKNTIVTSYNR
A_HCD
A_ETD
B_HCD
B_ETD
cross−linker cleavage
B+XL_L
B+XL_S
A+XL_S
A+XL_L
y10
y9
y8
c12
c11
z12
c10
z10
c9
c8
c6
c5
z9
b9
b8
z7
z6
y6
z5
c5
z4
b5
z3
b4
b3
c3
z2
b2
c2
z1
intensity
mass
0
50000
100000
150000
500
1000
1500
2000
2500
IQEAGTEVVKAK+XL_S
A_b_ion
A_y_ion
prec
y10+
y9+
y8+
y7+
y6+
y5+
y4+
y3+
y2+
b2+
b3+
b7+
b9+
b10+
b11+
y11++
intensity
m/z
IQEAGTEVVKAK+XL_L
A_b_ion
A_y_ion
prec
y10+
y9+
y8+
y7+
y6+
y4+
y3+
y2+
b2+
b3+
b5+
b7+
b8+
b9+
b10+
b11+
y11++
intensity
m/z
KGEKNTIVTSYNR+XL_S
B_b_ion
B_y_ion
prec
y5+
y6+
y4+
y3+
y2+
y7+
y8+
y9+
y10+
y11+
y12+
b4+
b5+
b6+
b7+
b8+
b10+
b11+
intensity
m/z
KGEKNTIVTSYNR+XL_L
B_b_ion
B_y_ion
prec
y4+
y3+
y7+
y8+
y9+
y10+
y11+
y12+
y10++
b3+
b7+
b8+
b10+
intensity
m/z
